# Enhanced Formation of Higher Fullerenes and Fullertubes: Aluminum Carbide Co-Vaporization with Graphite

**DOI:** 10.1021/jacsau.6c00922

**Published:** 2026-09-02

**Authors:** James C. Duchamp, Jaden Chin, Reonna Purtlebaugh, Yevva R. Ireland, Emily Nieting, Steven Stevenson, Harry C. Dorn

**Affiliations:** † Department of Physical Science and Engineering Science, 3217 Emory and Henry University, Emory, Virginia 24327, United States; ‡ Department of Chemistry, Virginia Tech, Blacksburg, Virginia 24060, United States; § Department of Chemistry and Biochemistry, 14688 Purdue University Fort Wayne, Fort Wayne, Indiana 46805, United States; ∥ FIRST Molecules Research Center, Purdue University Fort Wayne, Fort Wayne, Indiana 46805, United States

**Keywords:** fullerenes, fullertubes, SWNTs, formation, isolation, Purification, carbon, graphite, allotropes, Nanotubular fullerenes, aluminum, and aluminum carbide

## Abstract

The synergistic formation of new forms of carbon by covaporization of graphite and certain transition metal catalysts is well documented. Over 30 years ago, it was reported that covaporization of carbon and cobalt leads to enhanced formation of singIe-walled carbon nanotubes (SWCNTs) with diameters of ∼1.2 nm. We now report that covaporization of graphite and Al_4_C_3_ in a Krätschmer fullerene generator provides 1–2 orders of magnitude increase in the corresponding extract yields of higher spherical and elliptical fullerenes covering the range of C_100_ to C_240_. In addition, covaporization of graphite and Al_4_C_3_ also lead to significant increases in “fullertubes”. These molecules have tubular central section motifs equivalent to single walled carbon nanotubes, SWCNTs. These fullertubes exist as two prominent series, C_60_ + 10_*n*_: C_90_, C_100_, C_120_, C_130_, C_150_, and C_180_ and a series C_60+18n_: C_114_, C_132_, C_150_, C_168_, C_186_, and C_204_. As recently established, we employ an aminopropanol chemical reactivity protocol to establish the higher yields for these fullertubes. The higher fullerenes and fullertubes represent new carbon allotropes. Co-vaporization process provides an avenue for greater abundance of these large allotropic carbon molecules for various applications.

## Graphical abstract

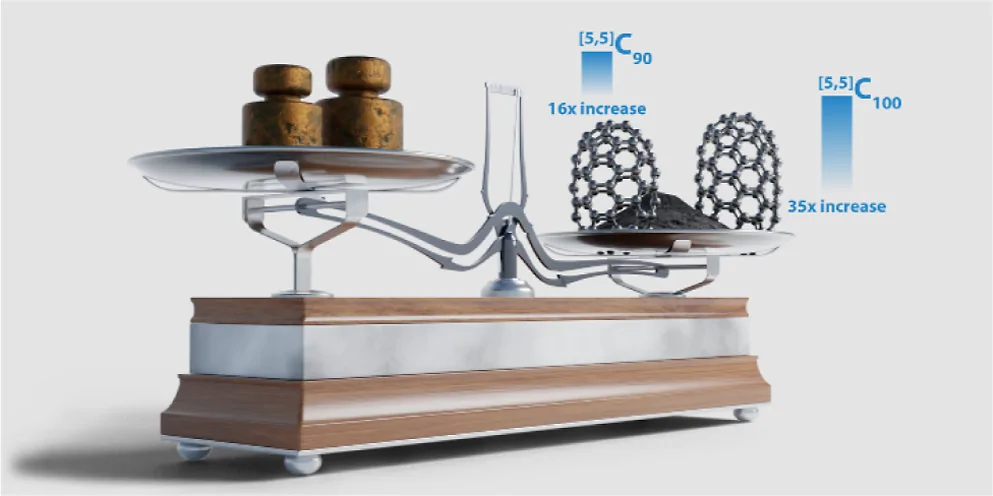

## 1 Introduction

In 1985, Smalley, Curl, and Kroto reported the discovery of a new allotrope of carbon, the fullerene C_60_, a high I_h_ symmetry molecule.^1^ It was quickly discovered that there exists a plethora of other higher fullerenes, but isolation and structural characterization of those above C_100_ are significantly hampered by a number of factors. First, although there is only one isolated pentagon rule (IPR) isomer for C_60_, the number of IPR isomers rapidly rises, 24 for C_84_ and 450 for C_100_.^2^ Furthermore, as the number of possible isomers increases, chemical structural differences are attenuated for a given mass. This gargantuan number of candidate isomers, along with HPLC coelution, represents a separation nightmare for isolating pristine isomers of any given mass above C_100_.

**Figure 1:**
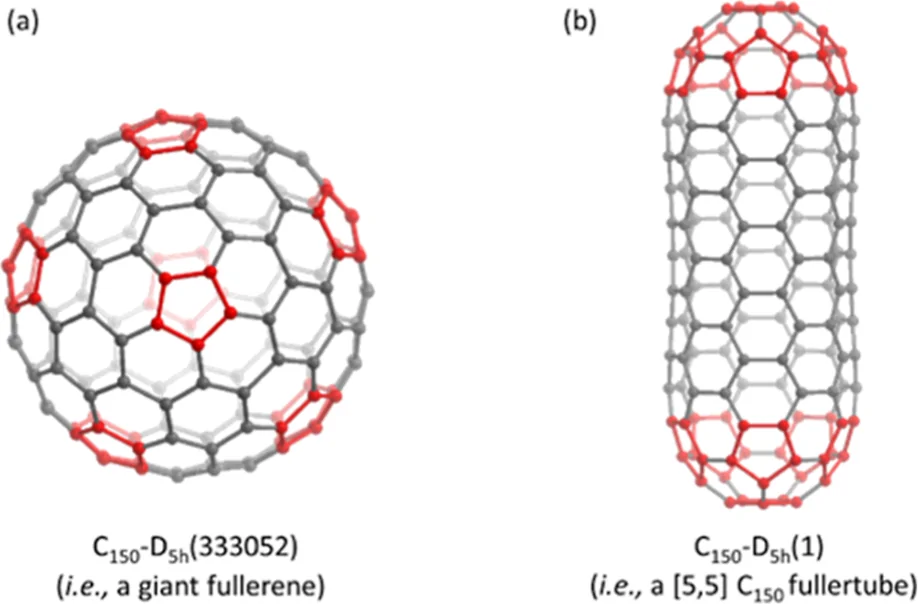
Representative giant fullertubes and fullerenes.

Another difficult issue for determining the structure of any fullerene over C_100_ is that the experimental isolation yields tend to dramatically decrease for fullerenes over C_100_. A third problem originally envisioned was anticipated lower solubility of these large molecules in organic solvents (toluene). However, this does not appear to be a severe limitation, except for some of the higher fullerenes. For illustrative purposes, representative structures for a 150-atom carbon cage of a high-symmetry fullerene (Figure 1a) and fullertube (Figure 1b) are provided to show differences in molecular shape.

Several years after the discovery of fullerenes, Bethune and Iijima independently discovered that after vaporization of graphite in a Krätschmer fullerene generator, another allotrope of carbon, single-walled carbon nanotubes (SWCNTs), was formed.^3,4^ A (*n*,*m*) indices descriptor has been employed to describe the length and circular vector for the hexagon sheet before roll-up as a (SWCNT). Specifically, most zigzag SWNTs (*n* > 0, *m* = 0) are semiconducting, but armchair SWNTs (*n* = *m*) are expected to exhibit metallic behavior. The carbon SWCNTs contain 1-d belts of sp^2^ hybridized tubular carbon that are structurally similar to fullertubes, but have open ends, vide infra. The SWCNTs have been extensively studied because of the immense number of potential applications of these 1-D nanomaterials. However, the same difficulty has occurred with SWCNTs as higher fullerenes, that is, the ability to obtain pristine samples of a single chirality has remained elusive.

In 2019, using the reactor design shown in Figure 2, soot was created that provided experimental proof of fullertube families that were discovered by Stevenson and reported at an ACS conference in Savannah, GA.^5^ These new allotropes of carbon link moieties of both SWCNTs and fullerenes. Specifically, the π-conjugated hexagon tubular belts of fullertubes are present for both SWCNTs and fullertubes, but the fullertubes have symmetry centered hexagon and pentagon fullerene C_60_ hemispheres as end-caps (Figure 1b). Note that a key difference between fullertubes and SWCNTs is the solubility of pristine fullertubes in various organic solvents, (CS_2_, toluene, chlorobenzene) without need for functionalization or polymer wrapping. Moreover, fullerenes are molecules of a fixed and known molecular mass. In contrast, nanotubes are neither molecules nor possess a reproducible and known molecular weight.

**Figure 2:**
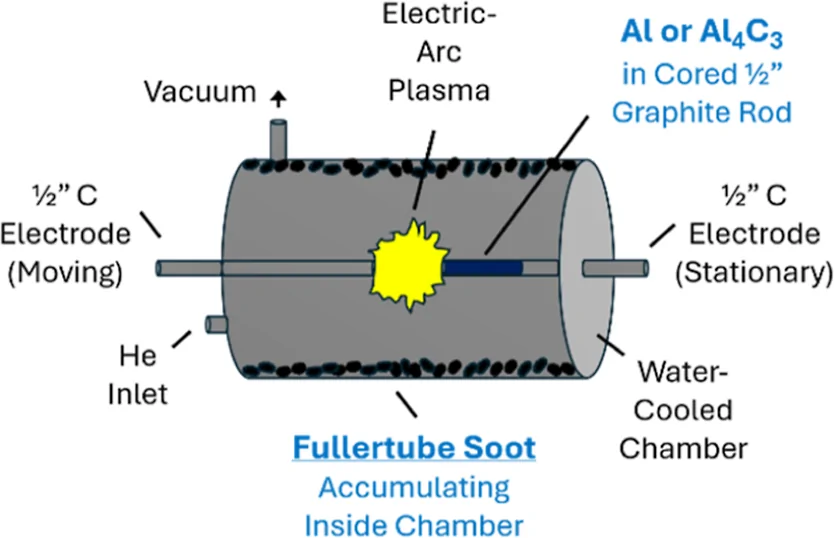
Electric-arc reactor and synthesis of fullertube soot.

Although an exact model for the growth of fullertubes from elementary atoms and molecules is not known, we envision a model similar to the model proposed by Dai and associates^6^ for the growth of SWCNTs and fullertubes from the surface of a metal cocatalysts as pictorially described in Figure 3. In the Dai model,^6^ the growth of SWNTs has a feature of a “dome-closed end”, which is the same molecular feature of the end-caps in fullertubes. The molecular fuel for the growth process from the surface of the metal particle is not totally understood and could involve C_2_ or even C atoms absorbed into the metal nanoparticle in a carbon–metal solid-state solution.

**Figure 3:**
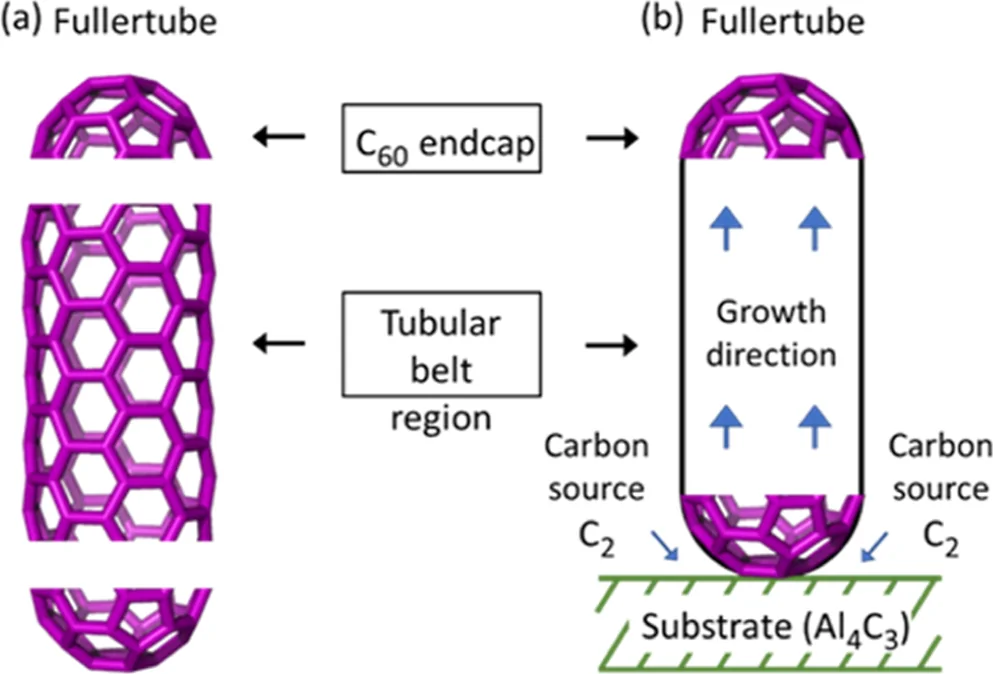
Dai model for SWCNTs applied to fullertubes.

For the case of the catalyst Al_4_C_3_, the molecular structure consists of isolated C^4–^ atoms that could be part of a catalytic atomic shuttle for growth of the fullertube or SWCNT. As the fullertube grows, a common feature for the fullertube formation is providing the same chirality at the top end-cap, which is removed from the metal catalyst surface. In any case, a common model may exist for the formation of both SWCNTs and fullertubes. However, even more complex moieties, such as C_60_ fragments, could be the starting building blocks at the metal catalytic surface, vide supra.

Since the discovery of C_60_ fullerene, the role of transition metals, e.g., Cu, Co, and Fe as cocatalysts in the preparation of higher fullerenes has been extensively explored.^7–12^ It should be noted, Bethune and co-workers discovered covaporization of graphite with cobalt^3^ in a Krätschmer fullerene generator which led to the discovery of SWCNTs. For example, Stevenson and co-workers reported higher yields of metallofullerenes in a Krätschmer fullerene generator with a copper metal powder additive to cored graphite rods.^11,12^ More recently, we reported higher yields of the higher C_90_ and C_100_ fullertubes when a metallic catalyst combination of Cu and Sc_2_O_3_ mixture was added to the graphite powder of packed carbon rods in a Krätschmer fullerene generator.^13^ Although it is well recognized that Sc when covaporized graphite forms Sc endohedral metallofullerenes, the other surprising result was higher yields of C_90_ and C_100_ fullertubes also formed when Cu was a plasma additive.

Nevertheless, there is a paucity of reports of other metals or nonmetals as possible cocatalysts in the Krätschmer fullerene generator for the formation of fullerenes. A notable exception is the report of SiC as a cocatalyst to form higher fullerenes C_76_, C_78_, C_80_, C_82_, and C_84_, but this report^14^ does not discuss the possibility of higher yields above C_100._ A related example is the report of preparing an exohedral functionalized derivative, C_60_H_2_-(C_3_H_5_N), in an electric arc synthesis using as a cocatalyst Al_4_C_3_ in the presence of NH_3_.^15^ The authors conclude that this study opens a new synthetic route to exohedral derivatized fullerenes using the reactive gas, NH_3_, and including Al_4_C_3_ as a reaction agent to produce in situ hydrocarbons.

## 2 Results and Discussion

Our initial investigation confirmed the enhanced yields of C_100_ when only Cu metal as well as other metal catalysts are covaporized with graphite as previously reported^11^ for Cu (See Supporting Information for further detail). Specifically, results indicate that Al metal powder catalyst was over 3× superior to the Cu at each of their optimum weight percentages of catalyst. In the current study, we further address the issue of improving experimental yields of fullerenes and fullertubes using Al and Al_4_C_3_ as cocatalysts to synthesize giant fullerenes (C_150_ and higher). As illustrated in Figure 4, the comparison of the extract yields of the C_90_ and C_100_ in separate covaporization experiments of Al compared with a control experiment (without catalyst). Analysis of the fullertube yield in extracts with added catalyst are significantly higher, i.e., increase by factors of 16 for [5,5] C_90_ and 35 for [5,5] C_100_ when 20% Al/80% (wt.-wt.) graphite were added to cored 1/2″ rods. See Supporting Information for experimental details.

**Figure 4:**
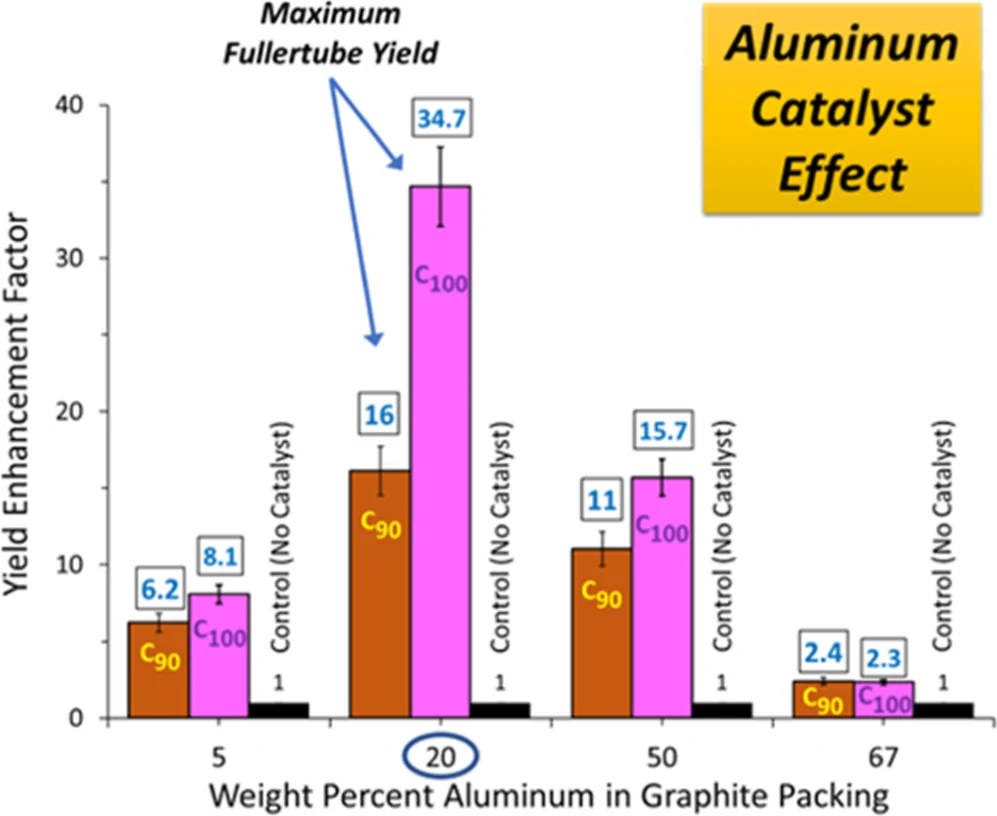
Yield of [5,5] C_90_ and [5,5] C_100_ fullertubes produced in soot extract obtained at 5, 20, 50, and 67% Al metal powder (wt.-wt.) added to graphite rods prior to arc-vaporization.

It is well recognized that in the Krätschmer fullerene generator, the source of vaporized carbon is resistively heated graphite rods placed in an electric-arc. In preparation for vaporization, the rods are cored and packed with the appropriate metal/graphite mixture. For the case of Al, this leads to other possible Al metal catalyst particles during the molecular formation of fullerenes, fullertube, and SWNTs. For example, it is well-known that Al metal forms aluminum carbide, (Al_4_C_3_), in the presence of graphite at 1000 °C. To examine this possibility, we have conducted experiments with various forms of Al as illustrated in Figure 5b–d at the same 20% catalyst level. A significant finding of the Al catalysts in Figure 5 is the steady progression of increased levels of higher quantities in the soluble extract with Al_4_C_3_. As demonstrated in the Figure 5d, Al_4_C_3_ served as a very efficient catalyst in producing a carbon mass distribution approaching C_250._ This increased distribution of higher fullerenes is significantly better than Al metal (Figure 5c). These findings reflect only nonpolar solvent (CS_2_ or chlorobenzene) extracted fullerenes/fullertubes as this soluble moiety was plated for mass spectral analysis. SWCNTs are insoluble in these solvents.

**Figure 5:**
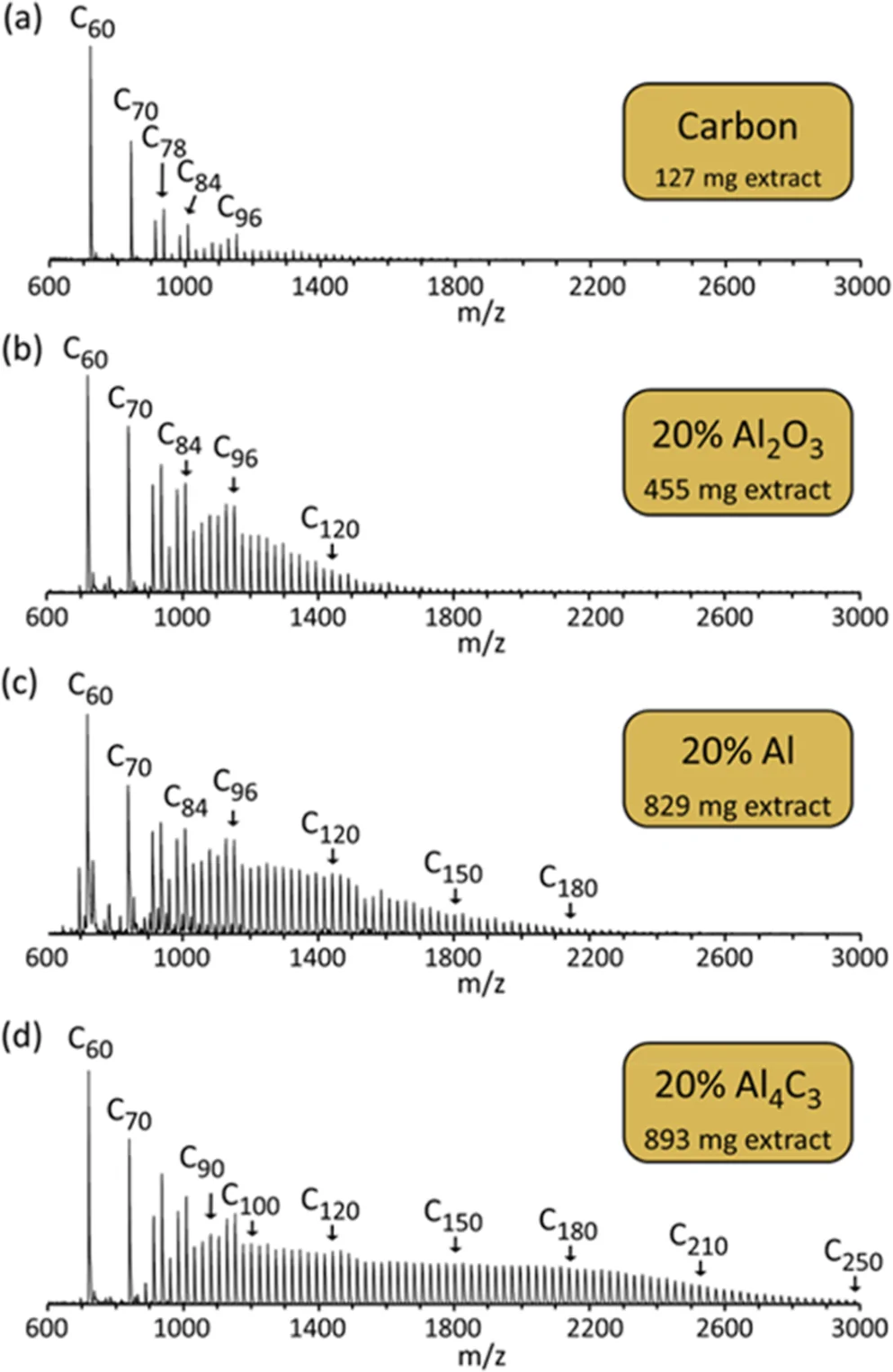
LDI mass spectra of chlorobenzene-extracted soot produced from (a) no catalyst compared to (b) 20% (wt.-wt.) Al_2_O_3_, (c) Al, or (d) Al_4_C_3_ packed graphite rods. Milligram quantities represent dried extract per rod (see Supporting Information).

As illustrated in Figure 6, covaporization of Al catalysts with graphite leads to increased yield of the less-studied C_74_ and C_80_ carbon cage structures in Krätschmer fullerene generator preparations. For example, there is the usual appearance of C_60_, C_70_, C_76_, C_78_, and C_84_, but there is also the strong appearance of C_80_, Figure 6b–d. To our knowledge, there is only a single report of isolation of the relatively unstable and pristine empty cage D_5d_-C_80_ fullerene by Shinohara and co-workers.^16^ A second uncommon fullerene is pristine C_74_. It should be noted that C_2_ extrusion and loss of C_2_ fragments can occur in mass spectral samples, we repeatedly noted that C_74_ and C_80_ peaks were consistently more abundant with the presence of aluminum-based catalysts. Slanina and co-workers^17^ have discussed the importance of this fullerene as an intermediate in the growth of other more common fullerenes. These results reflect the importance of Al and Al_4_C_3_ in the covaporization of carbon for preparation of new fullerenes and fullertubes.

**Figure 6:**
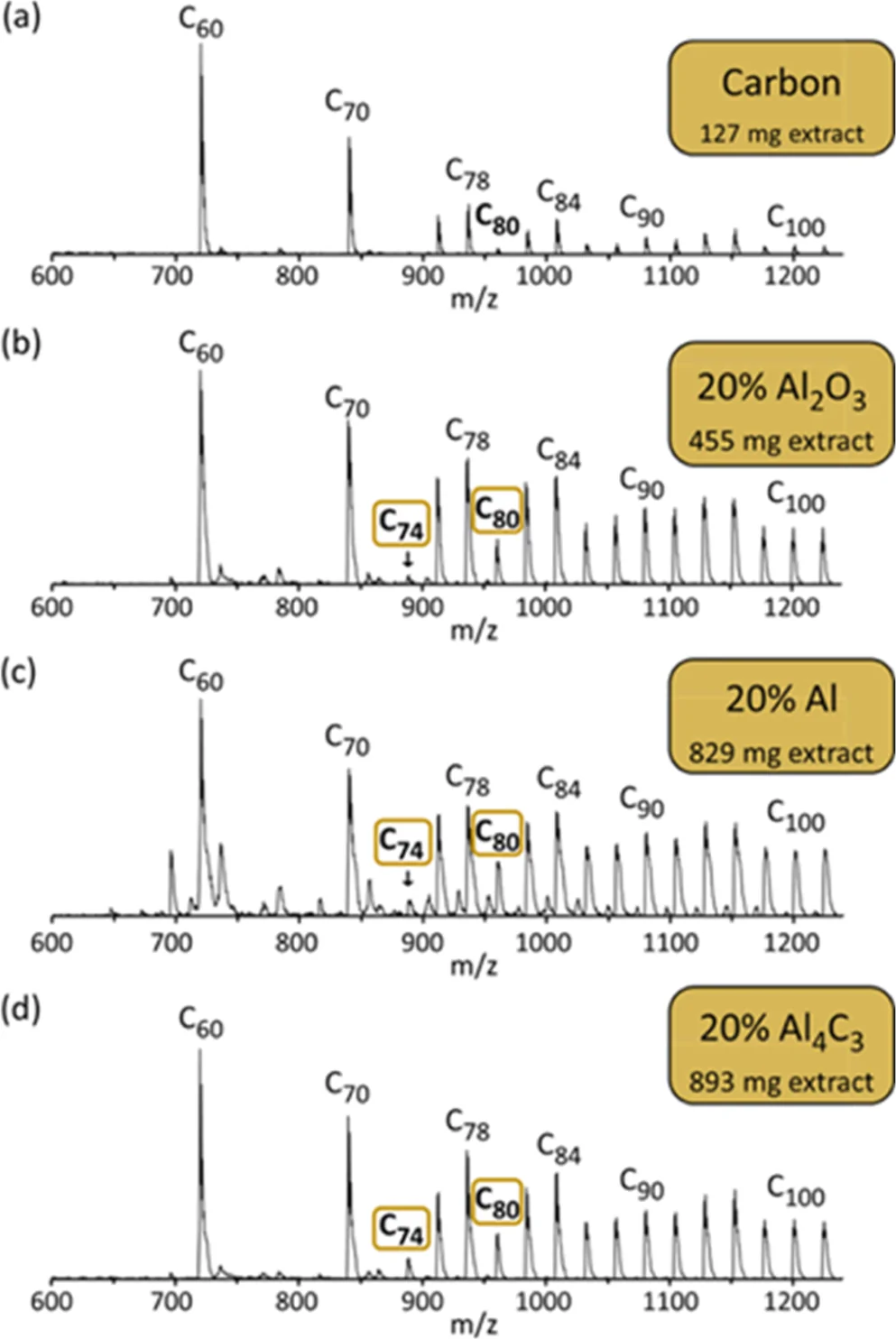
LDI mass spectra of lower fullerene (C_74_ and C_80_) distribution in soot extracts obtained from (a) no catalyst versus (b–d) various forms of Al covaporized with graphite. Milligram quantities represent dried extract per rod (see Supporting Information).

The discovery of a selective chemical reaction approach capable of distinguishing between spherical shaped fullerenes versus unreactive tubular shaped fullertubes was a major advancement.^18^ Specifically, the ability of aminopropanol to react selectively with spheroidal fullerenes in preference to less reactive elongated π tubular carbon molecules (i.e., fullertubes) represents an approach that is now well documented.^13,18–21^

In Figure 7, soot extract prepared from covaporization with various Al derivatives illustrates selective removal of spherical fullerenes after completing the aminopropanol precleanup protocol (Supporting Information). It is important to note that Al and Al_4_C_3_ significantly enhance the yields of the tubular fullertubes C_90_ and C_100_, Figure 7c,d. Moreover, there are two fullertube series readily apparent in Figure 7d. One is the readily recognized [9,0] C_96_, C_114_, C_132_, and C_150_ series originating from an end-cap symmetry 3-fold hexagon carbon ring carbon.^18^ The other readily recognized series is the end-cap pentagon [5,5] fullertube series, C_90_, C_100_, C_120_, C_130_, and C_150_.^13^

**Figure 7:**
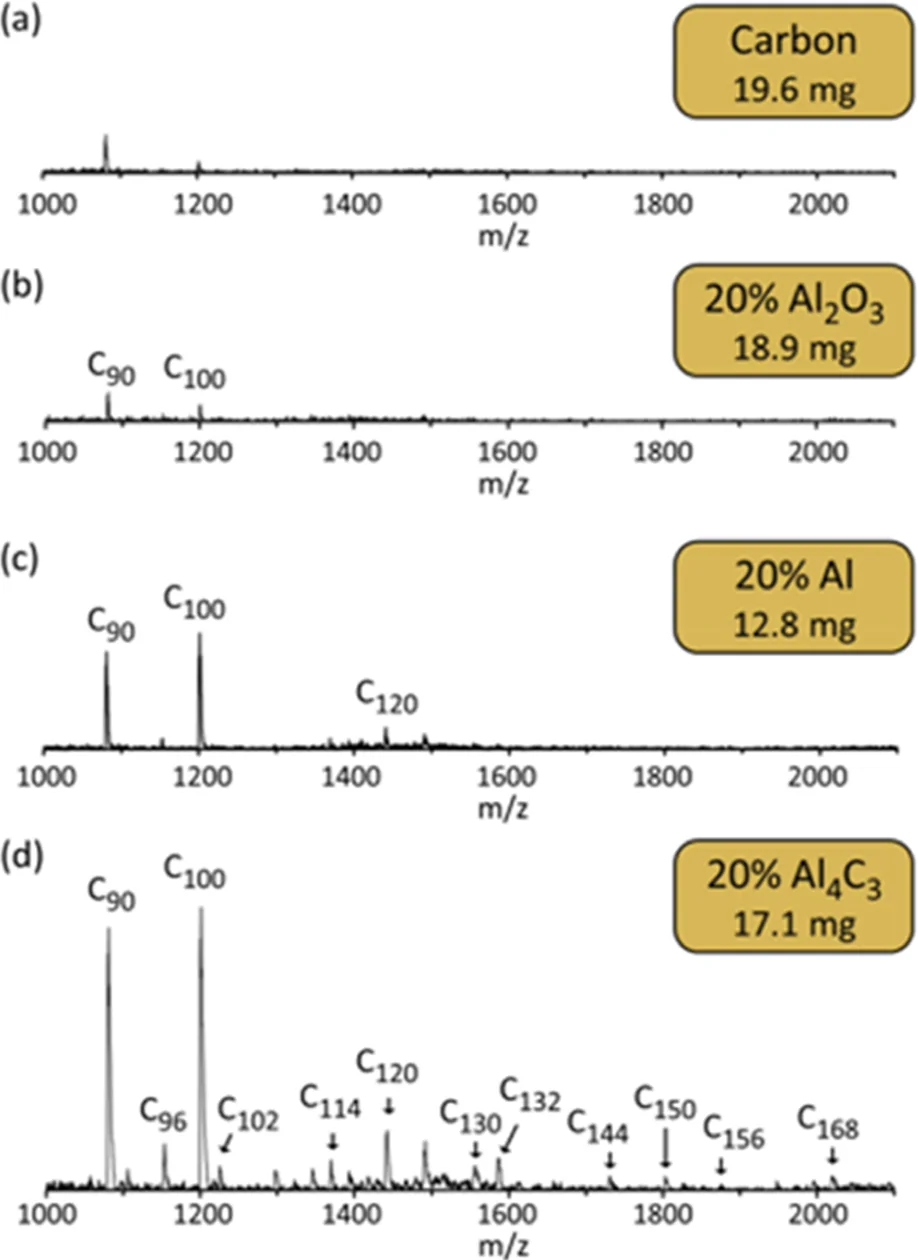
LDI mass spectra and mass of sample obtained after treatment of various soot extracts with aminopropanol to remove spheroidal fullerenes. (a) Control extract with no catalyst versus extract obtained from (b) 20% (wt.-wt.) Al_2_O_3_, (c) Al, or (d) Al_4_C_3_. Milligram quantities represent dried extract per rod (see Supporting Information).

For most covaporization experiments with transition metal cocatalysts conducted in Krätschmer fullerene generators, the optimum helium background pressure has usually been at ∼300 Torr. We have conducted experiments with the metal Al and the optimized extract yields also occur at ∼300 Torr as illustrated in the Supporting Information. However, the situation is different for Al_3_C_4_ where the high overall extract yield does not fall off as rapidly at higher helium pressures, Figure 8. There is also a significant increase in the quantity of higher range fullerenes with a maximum at C_120_ and extending to C_240_.

**Figure 8:**
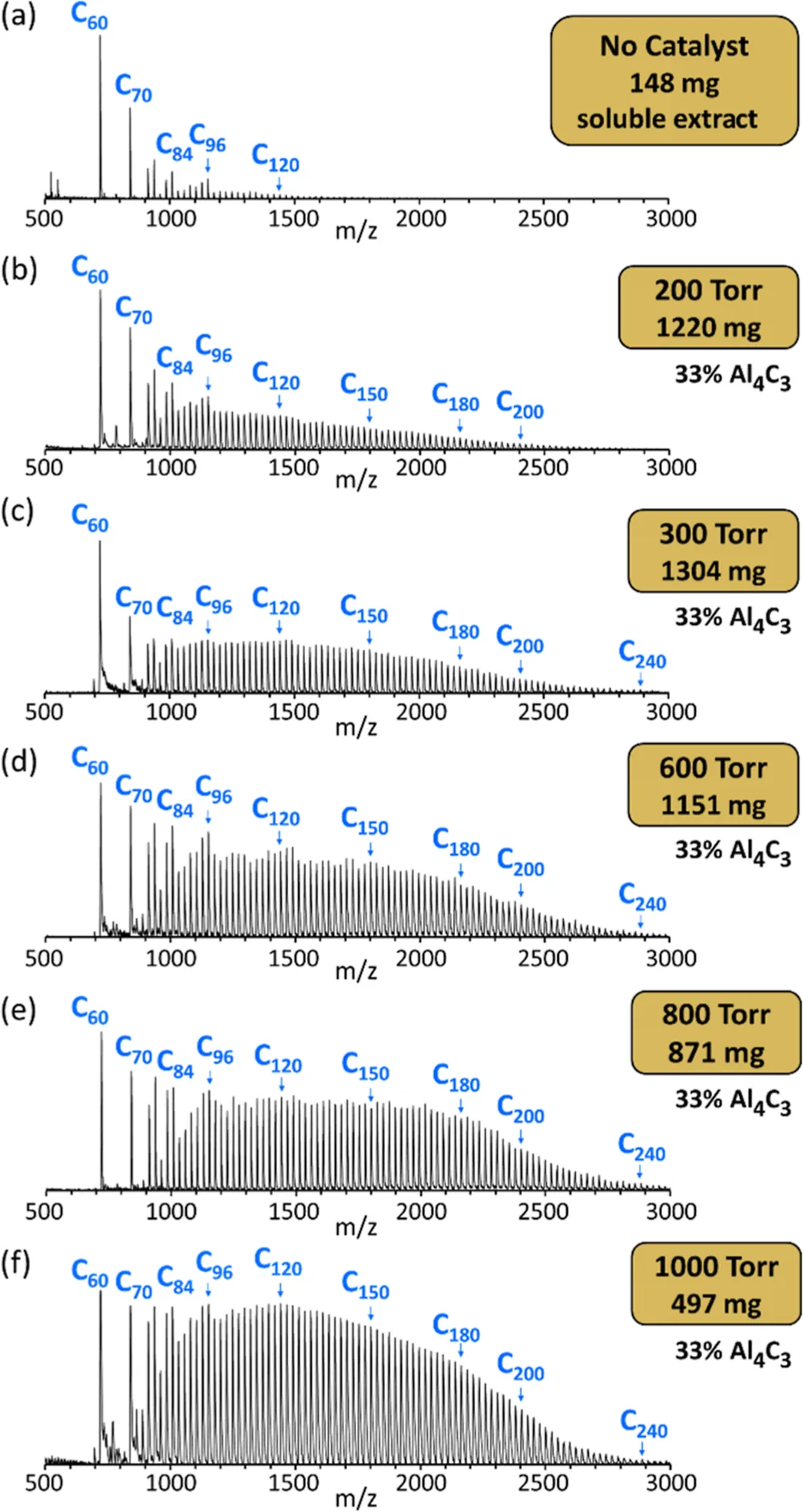
LDI mass spectra of extracted soot produced from (a) no catalyst added versus (b–f) various weight percentages of Al_4_C_3_ added to graphite for subsequent covaporization in the arc-reactor. Milligram quantities represent dried extract per rod (see Supporting Information).

More importantly, the yield of C_60_ significantly decreases in comparison to higher fullerenes (e.g., C_120_) as the helium buffer pressure increases from 200 up to 1000 Torr. One possible interpretation is that C_60_ fragments could be molecular building blocks at the metal catalytic surface for the formation of higher fullerenes with rising helium background pressure. In support of this argument, published work has described heating a fullerene C_60_ in the solid state at 800–1000° for 30–60 min under vacuum with a shift in the distribution toward larger masses.^22^ Moreover, it is reported in another paper that during space-tuned fullerene restructuring by calcining impregnated C_60_ in SiO_2_ nanovoids, the adjacent C_60_ fragments form sp^3^ bonding.^23^ In contrast, the unrestricted fragments without SiO_2_ confinement reconnect at the edges to form sp^2^-hybridized nanosheets.^23^ Both of these studies support the notion that C_60_ fragments could be molecular building blocks on metal catalytic surfaces for the formation of higher fullerenes at higher helium pressure in a Krätschmer fullerene generator vaporization of carbon.

For isolation of fullertube family members after successful chemical-based removal of soluble spherical fullerenes, the mass spectral profile for covaporization of carbon with Al_4_C_3_, Figure 9b, is directly compared with the mass spectral profile previously published findings without a catalyst used, Figure 9a.^20^ Although the two mass spectral profiles in Figure 9 appear similar, the amount of isolated soluble (CS_2_ or chlorobenzene) extract is at ∼2 orders of magnitude greater (factor of 86 increase) for the case of covaporization with Al_4_C_3_ catalyst. In addition, the concentration of C_90_ and C_100_ appears to significantly increase. Of the [5,5] series of fullertubes C_60_ + 10*n* (i.e., C_90_, C_100_, C_120_, C_130_, C_150_, and C_180_), only the first four have been reported and characterized.^13,18–21^ Structures of those four fullertubes are shown in Figure 9c. For the other series (i.e., C_96_ + 18*n*, C_114_, C_132_, C_150_, C_168_, C_186_, and C_204_), only the C_96_ fullertube has been characterized to date.^13,20,24^

**Figure 9:**
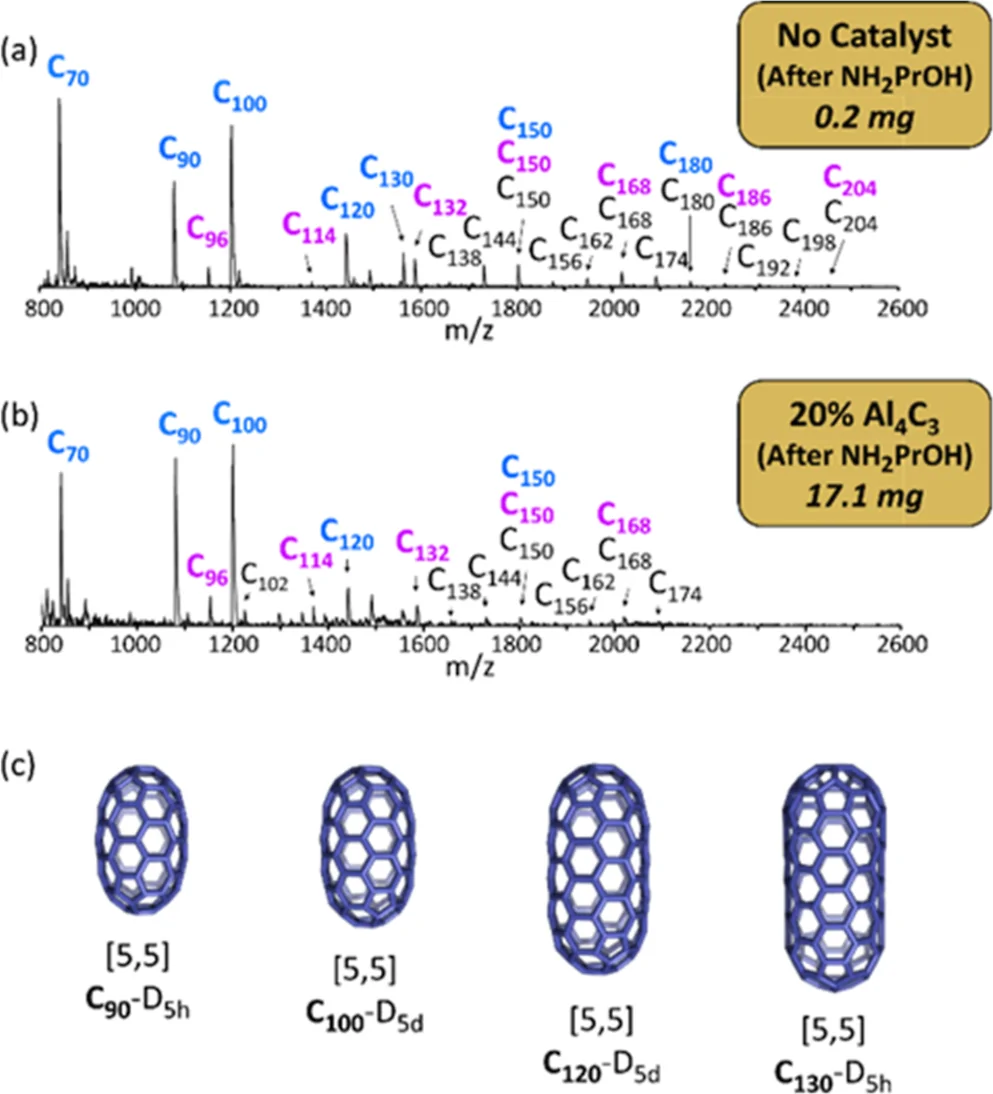
Comparison of (a) no catalyst versus (b) Al_4_C_3_ catalyst after treatment with aminopropanol to remove spheroidal fullerenes. (c) Structures of the four [5,5] fullertubes isolated to date. Milligram quantities represent dried material after chemical treatment with NH_2_PrOH (see Supporting Information).

## 3 Conclusions

From the results of Al_4_C_3_ covaporization, it is clear there is a large array of spherical fullerenes and fullertubes beyond C_100_ that could be isolated and characterized utilizing this new catalytic approach. In addition, this approach could lead to isolation of greater quantities of smaller fullerenes (e.g., C_74_ and C_80_) that could be isolated at higher extract levels. It should be noted that in the current study with the catalyst, Al_4_C_3_, we only considered organic solvent-soluble fullerenes and fullertubes. In this fashion, we have excluded consideration of the influence of Al_4_C_3_ on the yields of insoluble SWCNTs. The mechanistic molecular features for the formation of higher fullerenes and fullertubes with covaporization of Al_4_C_3_ exhibit unusual features including the possibility that C_60_ fragments could be building blocks for higher fullerenes well beyond C_100_.

Another unusual feature includes the mass spectral profile at elevated helium pressures, e.g., 1000 Torr in Figure 8f. Surprisingly, this 1000 Torr data compares favorably with the profile reported for the Murchison meteorite with a similar mass distribution approaching C_240_ and maximizing at ∼C_120_ (Figure 10).^25^

**Figure 10:**
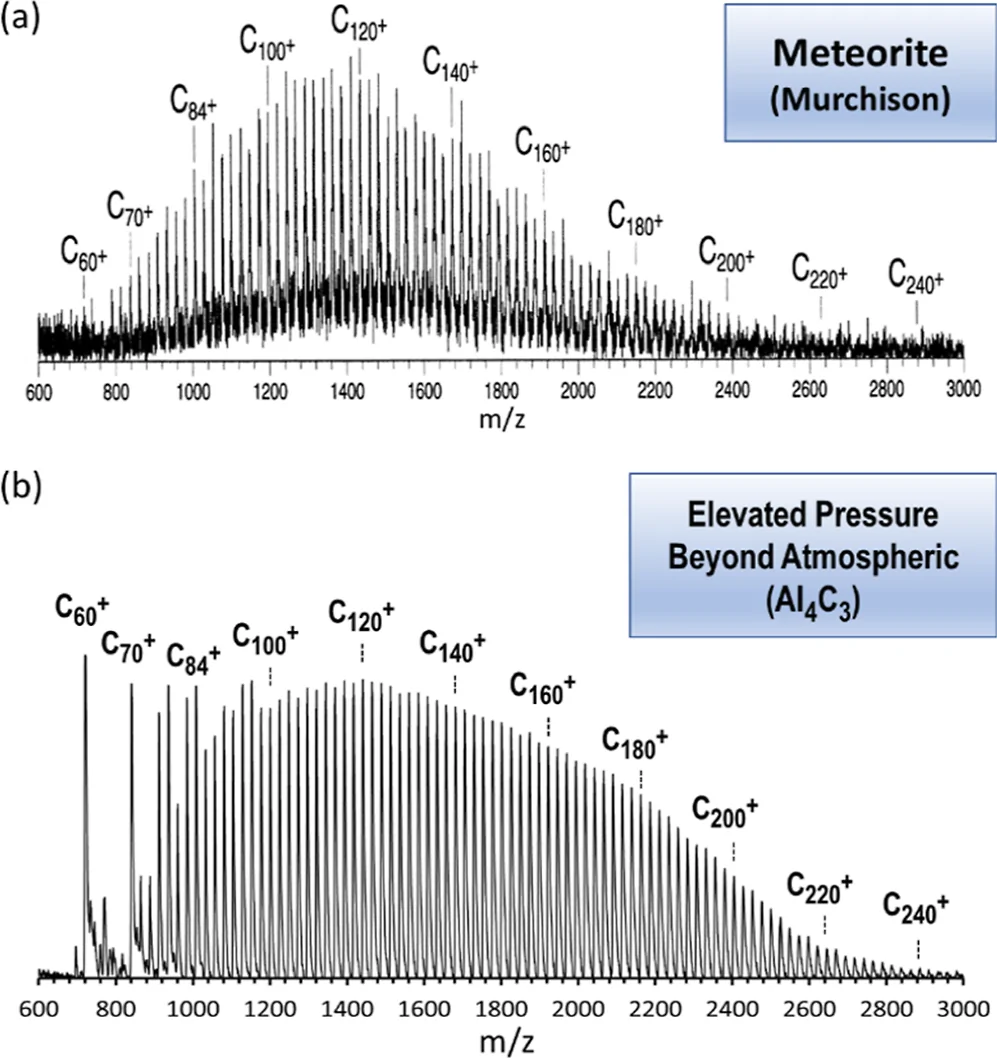
Comparison of LDI mass spectra obtained from the (a) Murchison meteorite in ref 25 versus (b) the extract herein obtained via high pressure arc-synthesis using Al_4_C_3_ catalyst to produce giant carbon cages.

## Supplementary Material



## Notes

Materials and methods; experimental details; analysis of fullertube content; electric-arc synthesis; and chemical isolation methodology (PDF)

## Notes

**In Memorial**: Harry Conrad Dorn was born on August 13, 1943, and died August 6, 2026. Of significance, this work represents Harry’s last scientific contribution. Harry deeply appreciated all his former students, collaborators, and family support throughout his fruitful career in science. Harry saw this JACS Au work through all phases of the publication process, e.g., research, writing, submission, response to reviewers, and revision. Harry literally worked on this paper until his final days. Since his arrival at Virginia Tech in 1974, Harry taught Chemistry and mentored the next generation of scientists. His early days at Virginia Tech focused on developing online HPLC-NMR detection and dynamic nuclear polarization. From 1990 to 2026, Harry contributed pioneering advances in fullerenes, metallofullerenes, and, most recently, fullertubes. He published many high impact papers in Nature, Science, Nature Chemistry, and JACS. His most published journal was JACS, with JACS Au being a fitting last publication as the Au gives the idea of a golden spirit in the golden era of science—a perfect summary of his career. Harry played a key role in the design and planning of the JACS Au supplemental cover art representing fullertubes and nanocarbons on earth and the cosmos.

## Abbreviations

IPR: isolated pentagon rule
LDI-TOF: laser desorption ionization—time-of-flight
HPLC: high performance/pressure liquid chromatography

## References

[1] Kroto H. W., Heath J. R., O’Brien S. C., Curl R. F., Smalley R. E. (1985). C60: Buckminsterfullerene. Nature.

[2] Fowler, P. W. ; Manolopoulos, D. E. An Atlas of Fullerenes; Clarendon, 1995.

[3] Bethune D. S., Kiang C. H., de Vries M. S., Gorman G., Savoy R., Vazquez J., Beyers R. (1993). Cobalt-catalysed growth
of carbon
nanotubes with single-atomic-layer walls. Nature.

[4] Iijima S., Ichihashi T. (1993). Single-shell carbon nanotubes of 1-nm diameter. Nature.

[5] Stevenson, S. Isolation of Tubular Carbon (FullerTubes): Fullerenes, Nanotubes, or Neither?. In ACS SERMAC Conference, 2019: Savannah, Georgia.

[6] Dai H. J. (2002). Carbon
nanotubes: Synthesis, integration, and properties. Acc. Chem. Res..

[7] Kikuchi K., Nakahara N., Wakabayashi T., Suzuki S., Saito K., Ikemoto I., Achiba Y. (1993). Higher Fullerenes - Separation and
Molecular Structures. Synth. Met..

[8] Liu X., Schmalz T. G., Klein D. J. (1992). Favorable
Structures for Higher Fullerenes. Chem. Phys.
Lett..

[9] Ahmad B., Riaz M., Ahmad M., Nawaz S., Ahmad S. (2008). Synthesis
and characterization of higher fullerene (C84) in dc arc discharge
using Cu as a catalyst. Mater. Lett..

[10] Grushko Y. S., Kozlov V. S., Artamonova T. O., Khodorkovskii M. A. (2012). Concentrating
of Higher Metallofullerenes. Fuller. Nanotub.
Carbon Nanostructures.

[11] Stevenson S., Mackey M. A., Thompson M. C., Coumbe H. L., Madasu P. K., Coumbe C. E., Phillips J. P. (2007). Effect of copper
metal on the yield
of Sc3N@C80 metallofullerenes. Chem. Commun..

[12] Stevenson S., Thompson M. C., Coumbe H. L., Mackey M. A., Coumbe C. E., Phillips J. P. (2007). Chemically adjusting plasma temperature, energy, and
reactivity (CAPTEAR) method using NOx and combustion for selective
synthesis of SC3N@C-80 metallic nitride fullerenes. J. Am. Chem. Soc..

[13] Stevenson S., Dorn H. C. (2024). Fullertubes: A 30-Year Story of Prediction, Experimental
Validation, and Applications for a Long-Missing Family of Soluble
Carbon Molecules. Acc. Chem. Res..

[14] Kareev I. E., Dutlov A. E., Bubnov V. P. (2020). Role of
Silicon Carbide in Arc Discharge
Synthesis of Higher Fullerenes. Technol. Phys..

[15] Chen N., Klod S., Rapta P., Popov A. A., Dunsch L. (2010). Direct Arc-Discharge
Assisted Synthesis of C60H2­(C3H5N): A cis-1-Pyrrolino C-60 Fullerene
Hydride with Unusual Redox Properties. Chem.
Mater..

[16] Wang C. R., Sugai T., Kai T., Tomiyama T., Shinohara H. (2000). Production
and Isolation of an Ellipsoidal C80 Fullerene. Chem. Commun..

[17] Slanina Z., Uhlik F., Zhao X., Adamowicz L., Nagase S. (2007). Relative Stabilities of C74 Isomers. Fuller. Nanotub. Carbon Nanostructures.

[18] Bourret E., Liu X., Noble C. A., Cover K., Davidson T. P., Huang R., Koenig R. M., Reeves K. S., Vlassiouk I. V., Côté M. (2023). Colossal C130 Fullertubes: Soluble [5,5]
C130-D5h(1) Pristine Molecules with 70 Nanotube Carbons and Two 30-Atom
Hemifullerene End-caps. J. Am. Chem. Soc..

[19] Liu X., Bourret E., Noble C. A., Cover K., Koenig R. M., Huang R., Franklin H. M., Feng X., Bodnar R. J., Zhang F. (2022). Gigantic C120 Fullertubes: Prediction and Experimental
Evidence for Isomerically Purified Metallic [5,5] C120-D5d(1) and
Nonmetallic [10,0] C120-D5h(10766). J. Am. Chem.
Soc..

[20] Koenig R. M., Tian H.-R., Seeler T. L., Tepper K. R., Franklin H. M., Chen Z.-C., Xie S.-Y., Stevenson S. (2020). Fullertubes:
Cylindrical Carbon with Half-Fullerene End-Caps and Tubular Graphene
Belts, Their Chemical Enrichment, Crystallography of Pristine C90-D5h(1)
and C100-D5d(1) Fullertubes, and Isolation of C108, C120, C132, and
C156 Cages of Unknown Structures. J. Am. Chem.
Soc..

[21] Stevenson S., Liu X., Sublett D. M., Koenig R. M., Seeler T. L., Tepper K. R., Franklin H. M., Wang X., Huang R., Feng X. (2021). Semiconducting and Metallic [5,5] Fullertube Nanowires: Characterization
of Pristine D5h(1)-C90 and D5d(1)-C100. J. Am.
Chem. Soc..

[22] Martin J. W., McIntosh G. J., Arul R., Oosterbeek R. N., Kraft M., Söhnel T. (2017). Giant fullerene formation through
thermal treatment of fullerene soot. Carbon.

[23] Li N., Guo K., Li M., Shao X., Du Z., Bao L., Yu Z., Lu X. (2023). Fullerene Fragment Restructuring: How Spatial Proximity
Shapes Defect-Rich Carbon Electrocatalysts. J. Am. Chem. Soc..

[24] Yang H., Jin H., Che Y., Hong B., Liu Z., Gharamaleki J. A., Olmstead M. M., Balch A. L. (2012). Isolation of Four Isomers of C96
and Crystallographic Characterization of Nanotubular D3d(3)-C96 and
the Somewhat Flat-Sided Sphere C2(181)-C96. Chem. - Eur. J..

[25] Becker L., Poreda R. J., Bunch T. E. (2000). Fullerenes:
An Extraterrestrial Carbon
Carrier Phase for Noble Gases. Proc. Natl. Acad.
Sci. U. S. A..

