# Supplementary material for: Enhanced Formation of Higher Fullerenes and Fullertubes: Aluminum Carbide Co-Vaporization with Graphite
Source: JACS Au. 2026 Sep 2;6(10):5641–7. doi: 10.1021/jacsau.6c00922 (PMC13714840; doi:10.1021/jacsau.6c00922)
Supplement: Supplementary file 1 [file au6c00922_si_001.pdf]

Supporting Information

Enhanced Formation of Higher Fullerenes and Fullertubes: Aluminum Carbide Co-vaporization with Graphite

James C. Duchamp[1,4*], Jaden Chin[3,4], Reonna Purtlebaugh[3,4], Yevva R. Ireland[3,4], Emily Nieting[3,4], Steven Stevenson*[3,4] and Harry C. Dorn*[2,4]

1) Department of Chemistry Emory and Henry College, Emory, Virginia 24327, USA

2) Department of Chemistry, Virginia Tech, Blacksburg, Virginia 24060, USA

3) Department of Chemistry and Biochemistry, Purdue University Fort Wayne, Fort Wayne, Indiana 46805, USA

4) FIRST Molecules Research Center, Purdue University Fort Wayne, Fort Wayne, Indiana 46805, USA

KEYWORDS Fullerenes, Fullertubes, SWNTs, Formation, Isolation, Purification, Carbon, Graphite, Allotropes, Nanotubular Fullerenes, Aluminum, and Aluminum Carbide.

Contents

1. Synthesis and Extraction of Fullertube Soot 1

2. Chemical Separation Procedure with Aminopropanol 2

3. Analysis of C_{90} and C_{100} Fullertube Content in Extract 3

4. Experimental Analysis of Soot Extract via MALDI-TOF 4

5. Optimal weight percent of catalyst added to graphite packing material 5

Material and Methods:

1. Synthesis and Extraction of Fullertube Soot

The electric-arc method was used to produce carbonaceous soot. Specifically, graphite rods of dimensions 6" in length and ½ "diameter graphite rods were core-drilled with a ¼" drill bit to a depth of 4 inches. Separately, a mortar and pestle were used to blend graphite powder with either aluminum metal, aluminum oxide, or aluminum carbide. These 325 mesh powder mixtures were

measured with weight/weight percentages, *i.e.*, 20 wt.% Al of packing material corresponded to a blended mixture containing 2 g Al and 8 g of graphite powder. Note: the shell graphite of the cored rods provides additional carbon available for the arc-plasma synthesis of fullerenes/fullertubes.

Each packed rod was loaded into the reactor for vaporization. A 2" long solid stub of a ½" diameter rod was placed into the opposite end of the chamber to complete the anode and cathode regions for the subsequent arc vaporization *via* a 500 A capacity Lincoln arc-welder. Experimental conditions were as follows: 260 A of current, a gap voltage of 30-36 V, and a He buffer gas under dynamic flow to achieve desired chamber pressures, *e.g.*, window of 75-1000 torr. Depending on the catalyst, weight percentage of added metal, gap voltage, length of packed rod that was vaporized, and/or He chamber pressure, the average burn times (*i.e.*, amount of time to vaporize the packed rod region) were approximately 35-50 minutes. Upon cooling the chamber, the produced soot (15-20 g) was collected. A mass spectral aliquot was obtained using carbon disulfide as an extraction solvent. The remaining soot (99.9%) was transferred to a 2L flask and immediately extracted using chlorobenzene to extract the fullerene/fullertube content. Extraction times were for 5 hours and included sonication and stirring. After solvent removal via rotary evaporator, the dried extract was then washed with diethyl ether to remove contaminant hydrocarbons prior to weighing.

2. Chemical Separation Procedure with Aminopropanol

As a pre-cleanup step to remove the bulk of spheroidal carbon, the extract obtained vide supra was subjected to the following procedure: 250 mg extract was placed in a 600 mL beaker and covered, placed on a stir plate and heated to a 31 ºC for 2 hours. With now vigorous stirring, 5 mL of aminopropanol was added to the stirring vortex, re-covered, and allowed to stir for 50 minutes of reaction time and removed from the hot/stir plate. After settling for an additional 50 minutes at room temperature, the reaction mixture (upper layer) was poured int a separatory funnel for 5 washes with deionized water, 1 time with 0.1 M HCl, and 5 additional washes with water. After rotary evaporation and ether washing of the dried powder, the milligram amounts were noted.

Aminopropanol was used the chemical reagent to selectively react and remove via a solubility change (hydrophobic to hydrophilic) contaminant spheroidal fullerene carbon cages (*e.g.*, C_{60}, C_{70}) away from the chemically resistant tubular carbon cages called fullertubes (*e.g.*, C_{90}, C_{96}, C_{100}, C_{120}). Upon chemical derivatization, the solubility of spheroidal species changes from organic soluble to water solubility. In contrast, tubular shaped carbon with fullerene endcaps (*i.e.*, fullertubes) possess a protective *tubular belt* of only hexagons (*i.e.*, no 5,6 reactive sites available) and are hence chemically resistant. As such, the fullertubes (tubular form with end capped carbon) remain largely unreactive and, hence, stay in the organic layer. Upon settling, now in 2 layers of solution (fullertubes in top layer of xylenes; reacted spheroidal fullerenes in the bottom layer, a simple draining of the hydrophilic layer easily removes the reacted spheroidal fullerene contaminants. Note: due to large amounts of C_{60} and C_{70} in the extracts, there remains unreacted C_{60}, C_{70} fullerenes leftover not having sufficient time to react.

3. Analysis of C_{90} and C_{100} Fullertube Content in Extract

For quantitative analysis of [5,5] fullertube content in each of these post-aminopropanol samples, an array of solutions was prepared at 5.0 mg/ 5.0 mL chlorobenzene (*i.e.*, HPLC solvent). For each chromatogram, 100 µL of PTFE syringe filtered samples were injected into a 4.6 mm x 250 mL PYE column (SES Research, Nacalai Tesque) at a flow rate of 0.6 mL/min mobile phase of chlorobenzene. Quantitation and yield enhancement factors were performed via integration of HPLC peak areas of [5,5] C_{90} and [5,5] C_{100} fullertube for comparison with other extracts and control samples.

To demonstrate the quantitation of C_{90} and C_{100} fullertube amounts in samples obtained from 20% aluminum, Supp. Fig. 1 shows chromatograms obtained after aminopropanol chemical pre-cleanup to remove empty-cage spheroidal fullerenes. It was necessary to react and remove co-eluting higher fullerenes that would otherwise complicate the chromatographic region where fullertubes elute in the 7 – 14 min. region. C_{90} and C_{100} elute at 7.88 min and 11.8 min. Further details of higher fullerene removal to permit simplified samples enriched in fullertubes are described in *J. Am. Chem. Soc.* **2020**, *142*, 15614–15623. Herein, we set the detector to 478 nm and subsequently measured the peak area of eluting [5,5] C_{90} fullertube at its known retention time and confirmed its C_{90} identity with its real-time elution through the flow cell using a PDA detector. The UV-vis spectrum for the eluting species was a match for the prior published UV-vis for standard samples of C_{90} fullertube, also in *J. Am. Chem. Soc.* **2020**, *142*, 15614–15623. Likewise, the C_{100} peak area integration and analysis were performed in a similar fashion, but using detection at 568 nm. (Note: C_{90} fullertube absorbs strongly at 478 nm; whereas, C_{100} fullertube has a strong absorption at 568 nm. This separate selection of wavelengths ensures that strong peak areas are obtained for injected samples containing C_{90} and C_{100} fullertubes. HPLC peak areas were compared with samples obtained from a carbon control (*i.e.*, no added catalyst) versus samples prepared from Al catalyst.

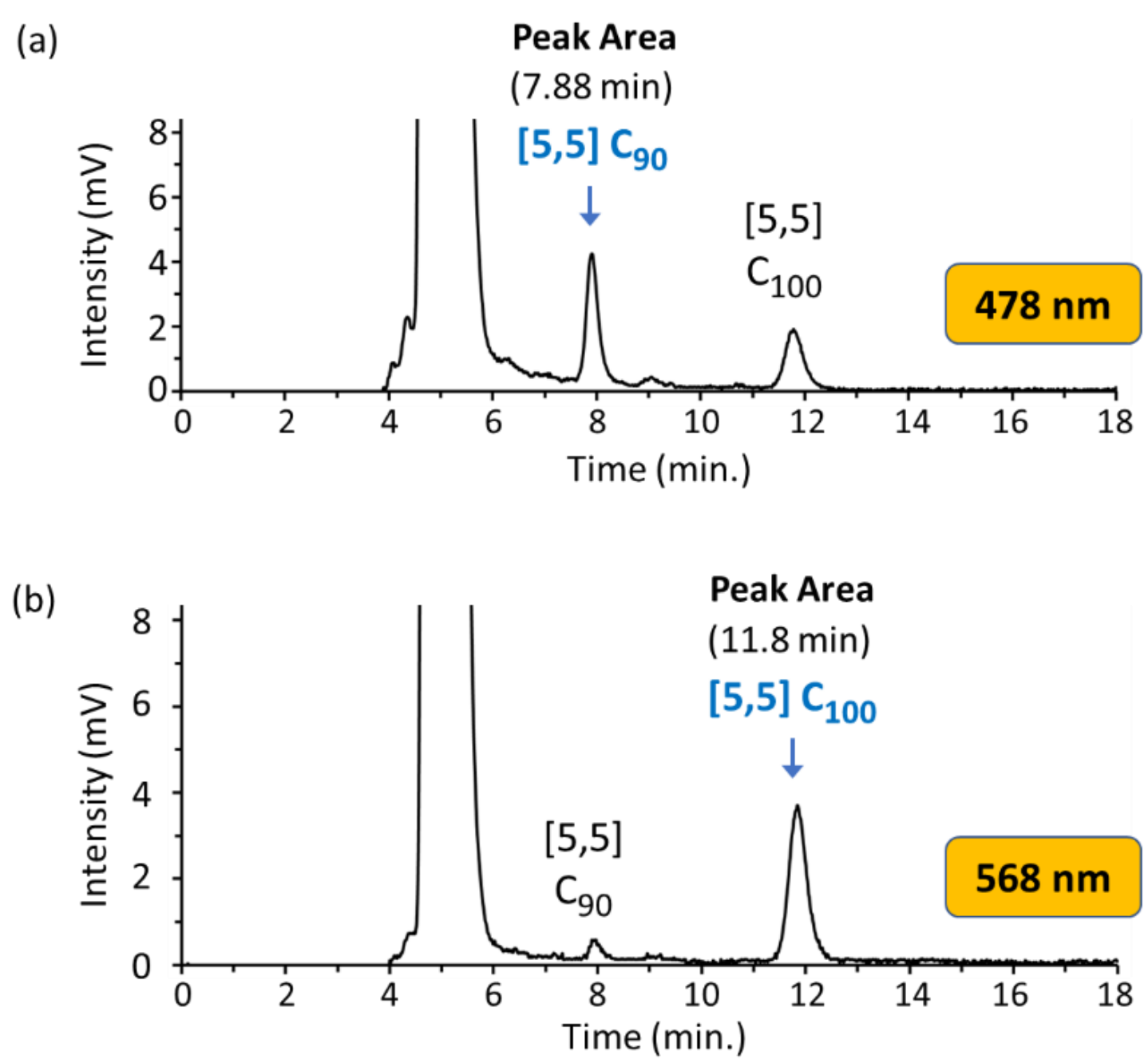

Supp. Fig. 1. HPLC chromatograms for 20% Al samples after aminopropanol pre-cleanup step and showing eluted peaks corresponding to (a) [5,5] C_{90} and (b) [5,5] C_{100} fullertubes.

In this manner, fullertube yields for soot prepared from no added catalyst (*i.e.*, C only in packed graphite rods) were successfully compared to extract where aluminum catalysts were utilized in soot production. A summary of these results is shown in Supp. Fig. 2.

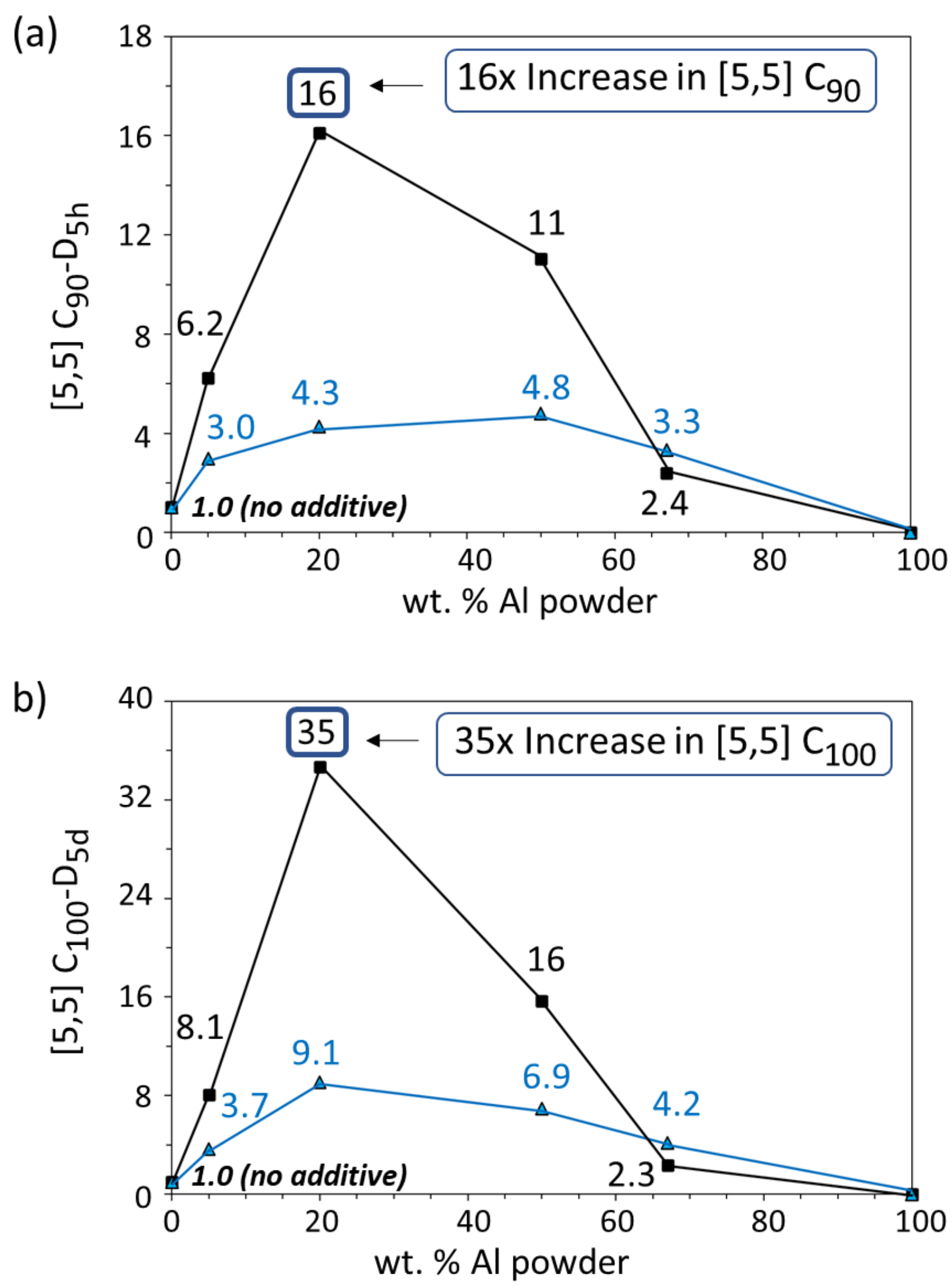

Supp. Fig. 2. Amount of (a) [5,5] C_{90} and (b) [5,5] C_{100} fullertubes present in soot extract versus extract obtained from a carbon control where no catalyst is added (*i.e.*, relative yield of 1.0)

4. Experimental Analysis of Soot Extract via MALDI-TOF

Supp. Table 1 summarizing the mass spectral data obtained from producing soot with Al versus Cu versus C control rods. The milligram amounts of dried extracts are provided, along with the composition of the packed rods of various weight percentages of aluminum or copper mixtures with graphite. The mass spectral intensity ratios of C_{90}/C_{70}, C_{100}/C_{70}, C_{120}/C_{70}, C_{150}/C_{70}, and C_{180}/C_{70} were obtained by collecting data from different sites on the stainless steel LDI target

place. This methodology allowed for standard deviation calculations for each of these ratios to C_{70} peak intensities.

Supp. Table 1. Summary of mass spectral data comparing extracts obtained from vaporization with Al and Cu catalysts versus control extracts with no added metal.

(a)

Al	mg soluble soot extract	Composition of packing material (wt-wt%)		90/70	100/70	120/70	150/70	180/70
	700.2	**5% Al** / 95% C	avg	1.002	1.002	1.080	0.756	0.695
			stdev	0.479	0.689	0.828	0.652	0.465
	1577	**20% Al** / 80% C	avg	1.035	1.470	2.253	2.270	2.038
			stdev	0.572	0.488	1.427	1.584	0.413
	1058	**50% Al** / 50% C	avg	0.554	0.359	0.272	0.151	0.090
			stdev	0.094	0.100	0.122	0.059	0.031
	454.4	**67% Al** / 33% C	avg	0.354	0.150	0.091	0.024	0.038
			stdev	0.079	0.040	0.030	0.010	0.018

(b)

Cu	mg soluble soot extract	Composition of packing material (wt-wt%)		90/70	100/70	120/70	150/70	180/70
	307.1	**5% Cu** / 95% C	avg	0.205	0.069	0.036	0.012	0.000
			stdev	0.058	0.023	0.013	0.002	0.000
	340.5	**20% Cu** / 80% C	avg	0.367	0.236	0.142	0.055	0.016
			stdev	0.069	0.047	0.026	0.011	0.004
	380.6	**50% Cu** / 50% C	avg	0.306	0.158	0.048	0.010	0.000
			stdev	0.078	0.039	0.008	0.002	0.000
	336	**67% Cu** / 33% C	avg	0.211	0.049	0.013	0.000	0.000
			stdev	0.035	0.007	0.003	0.000	0.000

(c)

C	mg soluble soot extract	Composition of packing material (wt-wt%)		90/70	100/70	120/70	150/70	180/70
	143.5	C Control	avg	0.162	0.038	0.019	0.009	0.000
		**Rod 1**	st.dev.	0.056	0.007	0.004	0.006	0.000
	109.4	C Control	avg	0.185	0.050	0.021	0.013	0.000
		**Rod 2**	st.dev.	0.064	0.017	0.008	0.006	0.000
	126.5	**Rods 1,2**	avg	0.174	0.044	0.020	0.0109	0.000
		**Rods 1,2**	st.dev.	0.005	0.007	0.003	0.0003	0.000

5. Optimal weight percent of catalyst added to graphite packing material

The optimal torr of helium pressure of aluminum packed rods was determined by analyzing the soot extracts after arc-synthesis done at a range of various chamber pressures. For aluminum metal packed rods, 300 torr of He provided the highest amount of extract (1540 mg) as shown in Supp. Fig. 3. A control rod vaporized with no added catalyst (*i.e.*, only carbon present) produced only

127 mg. This corresponds to an order of magnitude (12.7 times) increase in extract amounts with optimized aluminum metal conditions.

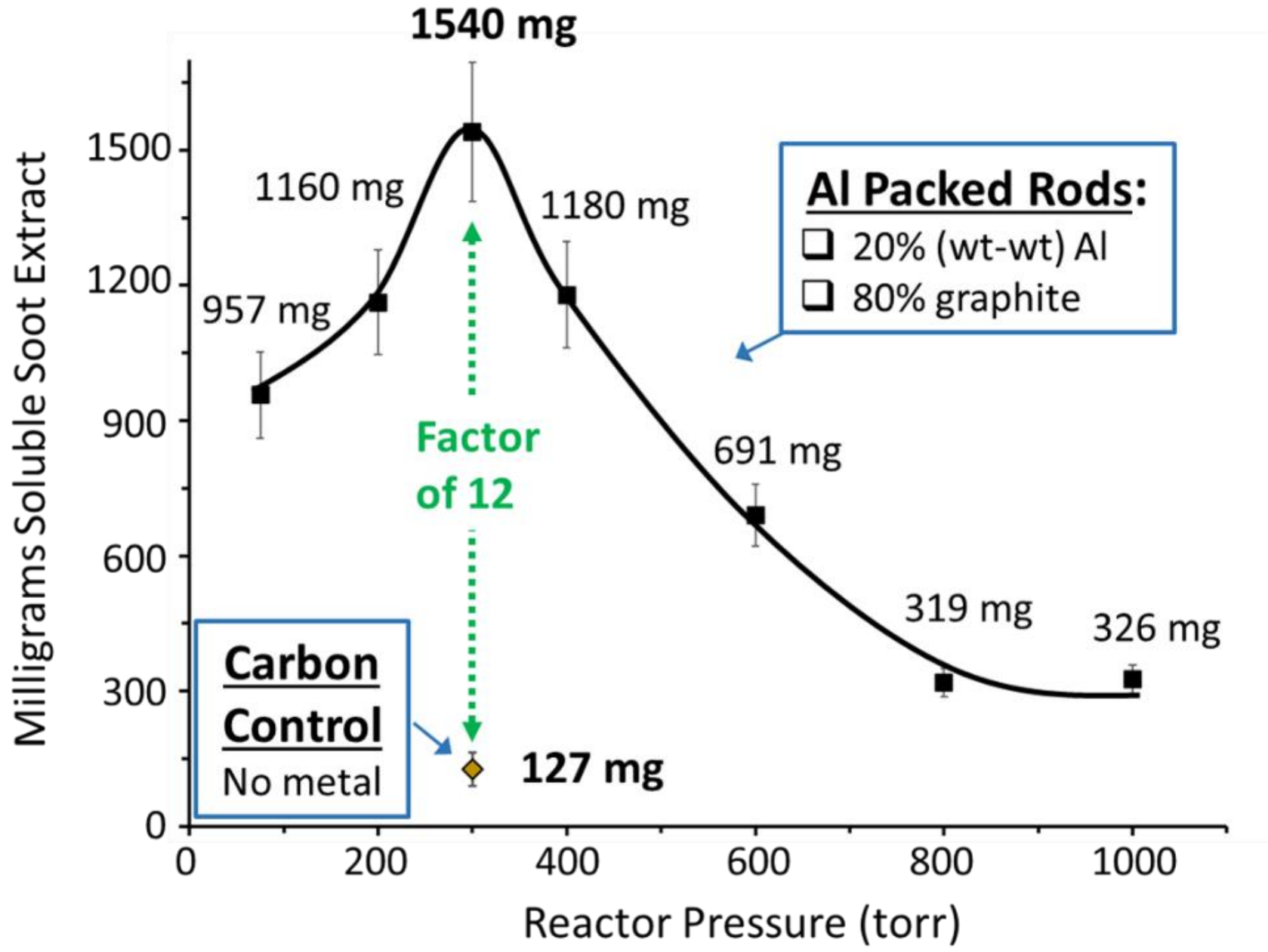

Supp. Fig. 3. Plot of milligrams extracted soot versus the chamber pressure of He gas
